# Clinical decision-making and health-related quality of life during first-line and maintenance therapy in patients with advanced non-small cell lung cancer (NSCLC): findings from a real-world setting

**DOI:** 10.1186/s12885-017-3543-7

**Published:** 2017-08-23

**Authors:** Monika Sztankay, Johannes Maria Giesinger, August Zabernigg, Elisabeth Krempler, Georg Pall, Wolfgang Hilbe, Otto Burghuber, Maximilian Hochmair, Gerhard Rumpold, Stephan Doering, Bernhard Holzner

**Affiliations:** 1 Department of Psychiatry, Psychotherapy & Psychosomatics, Medical University of Innsbruck, Innsbruck, Austria; 2 Leopold-Franzens-University of Innsbruck, Innsbruck, Austria; 3 Department of Internal Medicine, Kufstein County Hospital, Kufstein, Austria; 4 Waldburg-Zeil Akutkliniken GmbH & Co. KG, Wangen, Germany; 5 Department of Internal Medicine I (Haematology and Oncology), Wilhelminenspital Wien, Vienna, Austria; 6 Respiratory Oncology Unit, Department of Respiratory and Critical Care Medicine, Otto-Wagner-Spital, Vienna, Austria; 7 Department of Psychoanalysis and Psychotherapy, Medical University of Vienna, Vienna, Austria

**Keywords:** Non-small cell lung cancer, Pemetrexed, Maintenance therapy, Decision making, Health-related quality of life

## Abstract

**Background:**

Maintenance therapy (MT) with pemetrexed has been shown to improve overall and progression-free survival of patients with non-squamous non-small cell lung cancer (NSCLC), without impairing patients’ health-related quality of life (HRQOL) substantially. Comprehensive data on HRQOL under real-life conditions are necessary to enable informed decision-making. This study aims to (1) assess HRQOL during first-line chemotherapy and subsequent MT and (2) record patients’ and physicians’ reasons leading to clinical decisions on MT.

**Methods:**

Patients treated for NSCLC at three Austrian medical centres were included. HRQOL was assessed at every chemotherapy cycle using the EORTC QLQ-C30/+LC13 questionnaire. Semi-structured interviews were conducted before MT initiation and at the time of discontinuation to evaluate patients’ and physicians’ reasons for treatment decisions. Longitudinal QOL analysis was based on linear mixed models.

**Results:**

Sixty-one (73%) out of 84 patients were considered for MT. Thirty-six patients (43%) received MT and 29 (35%) discontinued therapy. Decisions on MT initiation (in 20 cases by the physician vs 4 by the patient) and discontinuation (19 vs 10) were mainly voiced by the physician. Treatment toxicity of first-line chemotherapy was the main reason for rejection of MT in patients with stable disease and was more often indicated by patients than clinicians. HRQOL data were collected from 83 patients at 422 assessment time points and indicated significantly lower symptom severity during MT compared with first-line therapy for nausea and vomiting (*p* = 0.006), sleep disturbances (*p* < 0.001), appetite loss (*p* = 0.043), constipation (*p* = 0.017) and chest pain (*p* = 0.022), and a deterioration in emotional functioning (*p* = 0.023) and cognitive functioning (*p* = 0.044) during MT.

**Conclusions:**

Our results indicate that HRQOL and symptom burden improve between first-line treatment to MT in some respects, although some late toxicity persists. Discrepancies between patients’ and physicians’ perception of reasons for rejecting MT were evident. Thus, the integration of patient-reported outcomes, such as HRQOL, is required to enable shared decision-making and personalised healthcare based on mutual understanding of treatment objectives.

## Background

Non-small cell lung cancer (NSCLC) accounts for 85% of all cases of lung cancer and causes the most cancer deaths worldwide [1, 2]. More than 50% of NSCLC patients present with advanced disease at diagnosis, for which four to six cycles of platinum-based doublet chemotherapy is the standard first-line treatment [3]. Patients responding to first-line therapy with stable disease or a partial/complete response after cycle 4 are considered for subsequent maintenance therapy (MT), either with a new agent (i.e. *switch MT*) such as pemetrexed or erlotinib or with one of the first-line agents (i.e. *continuation MT*) such as pemetrexed or bevacizumab [4, 5].

In clinical phase III trials, MT with pemetrexed has been shown to improve overall and progression-free survival of patients suffering from non-squamous NSCLC [6–8]. However, MT commits patients to continuous cytotoxic chemotherapy in a disease setting where the overall survival benefit remains modest [3, 9]. Hence, in line with European Society for Medical Oncology guidelines, decision-making about MT must take into account persisting toxicity after first-line chemotherapy, performance status and patients’ choices concerning treatment options [10]. In addition to physician ratings, toxicity can be comprehensively evaluated through patient-reported outcome measures (PROMs) such as the assessment of health-related quality of life (HRQOL).

Studies evaluating HRQOL in NSCLC clinical trials showed no substantial impairment of patients’ HRQOL following MT with pemetrexed [11–14]. Similar results have been reported for erlotinib [15, 16]. However, results on HRQOL from clinical trials should be interpreted with caution because of an inherent selection bias whereby patients with low income level or poor health status are less likely to participate in clinical trials, leading to the risk of overestimating HRQOL [17, 18].

To enable patients to make an informed decision on whether or not to undergo MT, both patients and physicians require comprehensive data on symptom burden and HRQOL under real-life conditions in this treatment setting [5]. Therefore, further investigation of HRQOL impairments related to pemetrexed MT has been encouraged [19, 20], especially in observational studies in a setting which considers the patient’s perspective. Therefore, the aim of this study was to assess HRQOL during first-line chemotherapy and subsequent MT, and determine patients’ and physicians’ reasons leading to clinical decisions in the treatment of advanced NSCLC.

## Methods

### Patients

Patients with advanced NSCLC were consecutively recruited at three Austrian medical centres (Otto-Wagner Hospital in Vienna, Medical University of Innsbruck and Kufstein County Hospital). Patients were eligible at the start of first-line palliative chemotherapy according to the inclusion criteria listed in Table 1.

**Table 1:** Inclusion and exclusion criteria

*Inclusion criteria*	diagnosis of NSCLC (adenocarcinoma or LC-anaplastic carcinoma)
	tumour stage IIIb (wet) or IV
	wild-type epidermal growth factor receptor (EGFR)
	first-line therapy with pemetrexed/platin or vinorelbine/platin
	MT with pemetrexed (in the case of remission or stable disease) or, alternatively, with erlotinib (only in the case of stable disease)
	aged between 18 and 90 years
	written informed consent
*Exclusion criteria*	obvious cognitive impairment

Sociodemographic and clinical data were collected from the medical charts.

### Ethics, consent and permissions

All participants provided written informed consent. The study was approved by the institutional review boards (Innsbruck Ethics Committee, reference number 4961).

### Assessment of patient choices and clinical decision-making

Clinical decision-making and patient choice concerning MT were assessed at the end of first-line palliative chemotherapy (T1) and, in the case of subsequent MT, at discontinuation of MT (T2). Both patients and physicians were interviewed using a semi-structured interview design with closed and open response formats.

Physicians were asked whether and what kind of MT (pemetrexed or erlotinib) they recommended for a specific patient. Where MT was not recommended, physicians were asked to provide the reason for this decision. Patients were interviewed concerning their decision to undergo MT or not, and the respective reasons for their decision. In the case of discontinuation of MT, the reason was assessed from the patient’s as well as the physician’s perspective.

### Health-related quality of life assessment

Patients’ QOL was assessed at each chemotherapy cycle (including MT) from the initiation of first-line palliative chemotherapy to the start of second-line palliative chemotherapy or at study completion (total of twelve assessment time points).

For HRQOL assessment, the EORTC QLQ-C30 [21] was applied, a widely used questionnaire for the assessment of QOL in cancer patients, and its lung-cancer-specific extension, the EORTC QLQ-LC13 questionnaire module. The EORTC QLQ-C30 covers five functioning domains (physical, role, social, emotional and cognitive), global QOL, eight symptoms (fatigue, pain, nausea/vomiting, appetite loss, insomnia, dyspnoea, diarrhoea, and constipation) and financial impact of the disease. The recently introduced QLQ-C30 summary score [22] aggregates all scales, except for global QOL and financial impact, into a summary measure of HRQOL.

The lung cancer-specific questionnaire EORTC QLQ-LC13 [23] assesses dyspnoea, coughing, haemoptysis, sore mouth, dysphagia, peripheral neuropathy, alopecia, chest pain, arm or shoulder pain, and other pain. It was supplemented with questions from the item library of the EORTC QOL Group to assess taste alterations and skin problems [24]. All questionnaire scales were scored 0–100, with high scores indicating good health status for functioning domains and poor health status for symptom domains.

Questionnaire assessments were performed electronically on tablet computers using the software CHES [25] (ESD, Innsbruck, Austria), which also provided the electronic case report forms used in this study.

### Statistical analysis

Results from the assessment of patient choices and clinical decision-making are provided as relative and absolute frequencies. The answers to open-ended questions were grouped into categories.

The analysis of HRQOL and symptoms was based on mixed linear models with questionnaire scales being the dependent variables and a time and treatment phase variable as fixed factors. In addition, the model included a diagonal covariance structure. Models were estimated separately for each of the questionnaire scales.

Mixed linear models are advantageous for this type of data as they allow the analysis of patients with different numbers of assessments as induced by attrition over time. We compared treatment phases (first-line chemotherapy vs. MT) and change over time within treatment phases using months since the start of treatment phase as the time variable. Since comparisons were only done over time (within-group comparisons) no covariates were included. Results are presented as estimated means and differences with their 95% confidence intervals.

All statistical analyses were performed using SPSS version 20.0 (IBM Corp. Released 2011. IBM SPSS Statistics for Windows, Version 20.0. Armonk, NY: IBM Corp.).

## Results

### Patient characteristics

Between March 2013 and July 2015, 87 patients were recruited for study inclusion (46 patients at Otto-Wagner-Hospital, 26 at Medical University of Innsbruck and 15 at Kufstein County Hospital). Three patients changed to a non-study centre during first-line chemotherapy and were excluded from the study. One patient did not provide HRQOL data. Thus, 84 patients were included in the analysis of clinical decision-making and 83 patients were included in the HRQOL analysis. Out of 84 patients, 47 (56.0%) were female. The mean age was 61.6 years (SD 9.8). In total, 27.8% of patients had undergone previous surgery and 10.1% had previously received radiation therapy.

Overall, 42.9% of first-line therapies were based on cisplatin and 44% on carboplatin (10.7% switched from cis- to carboplatin, 1.2% from carbo- to cisplatin, and 1.2% were treated with etoposide). The most common first-line chemotherapy regimens were combined pemetrexed and cisplatin (40.5%) and combined pemetrexed and carboplatin (33.3%). Nine patients (10.7%) started on pemetrexed and cisplatin and switched to pemetrexed and carboplatin. Twenty-two patients (26.2%) received three cycles or less, 57 patients (67.9%) received four cycles and five patients (6.0%) received five cycles or more. Further details are given in Table 2.

**Table 2:** Patient characteristics

Age	Mean (SD)	61.6 (9.8)	
		N	%
Sex	Women	47	56.0%
	Men	37	44.0%
Previous surgery	Yes	23	27.7%
	No	60	72.3%
	Missing	1	
Previous radiotherapy	Yes	8	10.1%
	No	71	89.9%
	Missing	5	
Regimens (1st line)	pemetrexed/cisplatin	34	40.5%
	pemetrexed/carboplatin	28	33.3%
	pemetrexed/ cisplatin (switch to pemetrexed/carboplatin)	9	10.7%
	vinorelbine/carboplatin	7	8.3%
	vinorelbine/cisplatin	2	2.4%
	pemetrexed/carboplatin, (second vinorelbine/carboplatin)	2	2.4%
	pemetrexed/carboplatin (reinduction with pemetrexed/cisplatin)	1	1.2%
	pemetrexed/etoposid	1	1.2%
Cycles	1 cycle	3	3.6%
	2 cycles	7	8.3%
	3 cycles	12	14.3%
	4 cycles	57	67.9%
	5 cycles	2	2.4%
	6 cycles	2	2.4%
	8 cycles	1	1.2%

### Clinical decision-making and patient choice

**Fig. 1:**
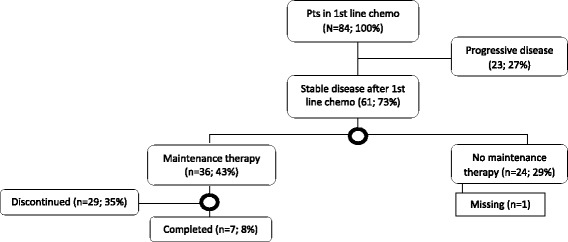
Patient distribution in treatment trajectory, circles indicating time point for interview on decision making. Data on one patient missing

Following first-line chemotherapy, 61 out of 84 patients (73%) were eligible for MT, whereas 23 patients (27%) had progressive disease. Among patients with stable disease or partial/complete remission after first-line chemotherapy, 36 (43%) received MT (33 patients received pemetrexed and 3 received erlotinib). Data on treatment status was unavailable for one patient. Twenty-nine out of 36 patients discontinued MT for reasons given below, while 7 patients were still on MT at study completion. Figure 1 presents the treatment trajectories.

#### Reasons for not undergoing MT in patients with stable disease or partial/complete response after first-line chemotherapy

Twenty-four patients with stable disease or partial response after first-line chemotherapy did not undergo MT. In most cases (20), the decision was made by the physician, mainly based on toxicity and side effects. Four patients declined MT with pemetrexed (3) or erlotinib (1), despite the physician’s recommendation to initiate MT. The main reasons indicated by patients for not opting for MT were the toxicity and side-effects of first-line therapy, the need for a treatment break and physical or emotional exhaustion (Table 3).

**Table 3:** Reasons for not undergoing or discontinuing MT (multiple reasons possible)

Reasons for not undergoing maintenance therapy (*n* = 24)		Reasons for discontinuation of maintenance therapy (*n* = 29)	
*PHYSICIAN*	*PATIENT*	*PHYSICIAN*	*PATIENT*
Toxicity or side-effects (*n* = 7)	Toxicity or side-effects (*n* = 14)	Disease progression (*n* = 18)	Disease progression (*n* = 9)
Regression/ curative treatment (*n* = 5)	Need for treatment break (*n* = 12)	Poor health status (*n* = 5)	Physical or emotional exhaustion (*n* = 6)
Patient wish for treatment holiday (*n* = 4)	Physical or emotional exhaustion (*n* = 10)	Treatment toxicity (*n* = 4)	Need for treatment break (*n* = 6)
Poor health status (*n* = 3)	Doubts that treatment would improve the condition (*n* = 7)	New comorbidities/ metastases (*n* = 2)	Treatment toxicity and side-effects (*n* = 5)
Patient compliance, disease progression (*n* = 2 each)	Reasons not specified (*n* = 4)	Remission or deceased (*n* = 1 each)	Time burden (*n* = 2)
New comorbidity, not eligible (*n* = 1 each)	Recommendation from family or friends (*n* = 3)		
	Financial and time burden (*n* = 2 each)		
	Curative treatment, poor health status (*n* = 1 each)		

#### Reasons for discontinuation of MT

Twenty-nine patients undergoing MT discontinued treatment. In the majority of cases (19), the decision not to continue with MT was made by the physician, while the decision was perceived as shared or was a direct decision by the patient in 5 cases, respectively. The main reason for discontinuation according to the physician’s decision was tumour progression (18), which resulted in a direct switch to second-line chemotherapy (e.g. docetaxel or erlotinib).

Patients’ reasons for not continuing MT included disease progression (indicated by 9 patients), physical and emotional exhaustion (6), need for a treatment break (6), toxicity or side-effects (5) and time constraints (2). Doubts about treatment efficacy, financial burden or the recommendation of family and friends were not reported to have affected the decision for discontinuation.

### Health-related quality of life during first-line and maintenance chemotherapy

We analysed the course of HRQOL across two treatment phases, first-line chemotherapy and MT. Analysis of first-line chemotherapy and MT were based on data from 83 patients, representing 422 assessments in total. Cross-sectional data indicated a statistically significant difference on the QLQ-C30 summary score (*p* = 0.048) and for specific domains. Lower symptom severity during MT compared with first-line therapy was found for nausea and vomiting (13.5 vs. 8.2 points, *p* = 0.006), sleep disturbances (39.1 vs. 21.4 points, *p* < 0.001), appetite loss (26.1 vs. 18.6 points, *p* = 0.043), constipation (17.3 vs. 10.5 points, *p* = 0.017) and chest pain (13.2 vs. 8.0 points, *p* = 0.022). In contrast, we found higher burden during MT compared with first-line therapy for alopecia (21.7 vs. 10.2, *p* < 0.001) and taste alterations (31.4 vs. 19.7, *p* = 0.004). For further details, see Tables 4 and 5.

**Table 4:** EORTC QLQ-C30 scores during first-line chemotherapy and during MT

	1st Line		Maintenance					
	Mean	95% CI	Mean	95% CI	Diff.	95% CI	*F*-value	*p*-value
Physical Functioning	73.5	70.7-76.3	73.8	69.1-78.6	−0.3	−5.8-5.2	0.014	0.906
Role Functioning	62.4	58.5-66.2	59.1	53.0-65.1	3.3	−3.9-10.4	0.817	0.367
Social Functioning	66.2	62.8-69.7	70.5	64.9-76.0	−4.2	−10.7-2.3	1.630	0.203
Emotional Functioning	65.0	62.1-67.9	68.9	64.0-73.7	−3.9	−9.5-1.8	1.826	0.178
Cognitive Functioning	80.0	77.4-82.6	82.8	78.5-87.1	−2.8	−7.8-2.2	1.194	0.276
Global Quality of Life	58.2	55.7-60.8	57.3	53.1-61.4	1.0	−3.9-5.8	0.156	0.693
Fatigue	45.8	42.4-49.2	41.2	35.7-46.8	4.6	−1.9-11.1	1.949	0.164
Nausea/Vomiting	13.5	11.0-16.0	8.2	5.3-11.1	5.3	1.5-9.1	7.663	**0.006**
Pain	27.0	23.6-30.3	24.4	18.8-30.0	2.6	−3.9-9.1	0.612	0.435
Dyspnea	29.6	25.8-33.3	35.9	29.9-42.0	−6.4	−13.4-0.7	3.202	0.076
Sleep Disturbances	39.1	35.1-43.2	21.4	15.7-27.1	17.8	10.8-24.7	25.480	**<0.001**
Appetite Loss	26.1	22.2-29.9	18.6	12.5-24.7	7.5	0.2-14.7	4.172	**0.043**
Constipation	17.3	13.9-20.6	10.5	6.2-14.9	6.7	1.2-12.2	5.862	**0.017**
Diarrhea	7.8	5.4-10.3	6.1	2.5-9.7	1.7	−2.6-6.1	0.627	0.430
Financial Impact	19.6	16.3-23.0	23.6	17.9-29.3	−4.0	−10.6-2.6	1.426	0.234
QLQ-C30 Summary Score	71.8	69.7-73.9	75.7	72.4-79.0	−3.9	−7.9-0.0	3.948	**0.048**

*95% CI* 95% confidence interval; Bold type indicates *p* <.05

**Table 5:** EORTC QLQ-LC13 and other symptom scores during first-line chemotherapy and during MT

	1st Line		Maintenance					
	Mean	95% CI	Mean	95% CI	Diff.	95% CI	*F*-value	*p*-value
Dyspnoea	23.0	19.9-26.0	21.2	0.0-45.4	1.73	−22.6-26.1	0.230	0.632
Coughing	31.1	27.8-34.4	26.7	21.4-32.0	4.4	−1.8-10.6	1.943	0.165
Haemoptysis	2.8	1.1-4.5	<0.1	0.0-10.5	2.8	−7.8-13.4	0.273	0.603
Sore mouth	8.0	5.5-10.4	7.0	2.9-11.1	1.0	−3.7-5.8	0.183	0.670
Dysphagia	9.1	6.8-11.4	6.1	1.7-10.6	3.0	−2.0-8.0	1.408	0.237
Peripheral neuropathy	14.9	11.8-18.0	18.8	13.7-23.9	−3.9	−9.9-2.0	1.711	0.192
Alopecia	10.2	7.3-13.0	21.7	16.6-26.8	−11.5	−17.4- -5.7	15.116	**<0.001**
Pain in chest	13.2	10.8-15.7	8.0	4.3-11.7	5.3	0.8-9.7	5.437	**0.022**
Pain in arm or shoulder	18.1	14.8-21.3	19.6	14.6-24.7	−1.6	−7.6-4.4	0.266	0.607
Pain in other parts	24.9	21.0-28.9	25.2	18.3-32.0	−0.2	−8.1-7.6	0.004	0.951
Taste Alterations^a^	19.7	16.1-23.2	31.4	24.3-38.4	−11.7	−19.6- -3.7	8.442	**0.004**
Skin Toxicity^a^	18.1	15.0-21.3	16.9	12.0-21.8	1.2	−4.6-7.0	0.179	0.673

*95% CI* 95% confidence interval; Bold type indicates *p* <.05

^a^items from the EORTC item bank

Analysis of changes during first-line chemotherapy showed a statistically significant reduction in coughing (*p* = 0.035) and pain in the arm or shoulder (*p* = 0.023). In contrast, nausea and vomiting (*p* = 0.012), constipation (*p* = 0.003) and alopecia (*p* < 0.001) increased throughout first-line chemotherapy. During MT, emotional functioning (*p* = 0.023) and cognitive functioning (*p* = 0.044) deteriorated and appetite loss (*p* = 0.001), constipation (*p* = 0.003) and financial impact (*p* = 0.041) increased. Nausea and vomiting improved over time (*p* < 0.001), given the discontinuation of platin-based chemotherapy in MT. As a result of attrition, the longitudinal analysis only covered the first 4 months of each treatment phase, with later time points being excluded.

## Discussion

This study aimed at assessing clinical decision-making for MT with either pemetrexed or erlotinib as well as HRQOL in patients with advanced NSCLC in a real-world setting. Compared with first-line therapy, we found that the HRQOL and symptom burden improved in MT for symptoms such as nausea and vomiting, sleep disturbances, constipation and appetite loss. While this may be partly explained by the discontinuation of platin-based chemotherapy, the reported increase in alopecia and taste alterations might possibly indicate late first-line treatment toxicity.

Our findings are comparable to those of studies reporting on HRQOL in maintenance pemetrexed [11–13] showing that HRQOL seems to be at least maintained during long-term MT for certain symptoms while further impairing others. When compared with patients receiving placebo, patients undergoing MT with both pemetrexed [13] and erlotinib [15] reported similar HRQOL using the self-administered Functional Assessment of Cancer Therapy-Lung (FACT-L), with the exception of a larger degree of appetite loss under MT as well a significantly prolonged time to worsening of pain and analgesic use.

In the landmark PARAMOUNT trial [14], pemetrexed was associated with significantly more low-grade nausea, anaemia, oedema, and neutropenia than placebo, while the incidence of low-grade fatigue, anaemia, and neutropenia decreased with longer treatment exposure. Though HRQOL impairments in the course of long-term pemetrexed maintenance as measured by the EQ-5D were not substantial, even low-grade toxicities were reported to have been potentially burdensome for patients.

In our study, treatment toxicity and side effects as well as physical and emotional exhaustion were the most common reasons for patients to decline MT in the first place. The reported incidence of reasons, however, might imply that clinicians underestimate the effects of patient-reported toxicity and treatment sequelae. Treatment toxicity and side effects of first-line chemotherapy were indicated twice as often by patients than by physicians to be the reason for not considering MT, despite stable disease. Patients further reported physical or emotional exhaustion in 10 cases. This finding is consistent with other treatment preference studies [26] where the extent and severity of current treatment-related side effects played a large role in patients’ attitudes to continued treatment. While patients attach high value to delaying the worsening of symptoms [27], progression-free survival benefits are viewed as most beneficial when disease symptoms are mild but as detrimental when disease symptoms are severe [28].

This discrepancy in patients’ and physicians’ perceptions of treatment burden and reasoning for and against MT reflects the notion that information reported by the physician cannot substitute direct patient reporting [29]. In this study, the physician voiced the decision to opt for or discontinue MT in the majority of cases. This emphasises the need for closer integration of PROMs such as the assessment of HRQOL and patient choice of treatment into the process of clinical decision-making on MT. Systematic collection of patient-reported outcome data in the real-world clinical setting has proven feasible and can contribute to clinical management on different levels [30–32]. Group-level data on HRQOL during MT provide patients and clinicians with information to substantiate possible treatment-related functional and HRQOL consequences, thereby promoting shared and informed decision-making. On the individual patient level, continuous PROM monitoring supports personalised clinical management, enhancing patient–physician communication and continuity of care [33, 34].

The interpretation of our results is limited by the fact that while addressing first-line chemotherapy and MT, the study excluded second-line treatment as well as patients who did not receive MT after first-line chemotherapy. The latter, in particular, would have represented an interesting comparator group but were not assessable for logistical reasons. Because of the study sample size, particularly during MT, statistical power allowed only limited analyses of change over time. Belani et al. [13] reported similar problems regarding compliance with questionnaire completion in the PARAMOUNT trial. Despite the study limitations, however, the comprehensive assessment of HRQOL and symptom burden using the EORTC QLQ-C30, its lung cancer-specific modules QLQ-LC13, and additional items on taste alterations and skin toxicity are strengths of this study.

## Conclusions

Our results indicate that HRQOL and symptom burden during MT stabilise over time in some aspects, while remaining debilitating in others. Treatment burden dominates patients’ perspectives on therapy and affects their treatment decisions. Comprehensive data on symptom burden and HRQOL impact of MT systematically assessed under real-life conditions can contribute to optimised clinical care. As the use of MT is increasingly considered in other advanced cancers [35, 36], the integration of PROMs generated in clinical trials as well as on the individual patient level is required to enable shared decision-making and personalised health care based on a mutual understanding of treatment objectives and expectations.

## Abbreviations

EORTC QLQ-C30: European Organisation of Research and Treatment of Cancer Quality of Life Questionnaire Core 30
EORTC QLQ-LC13: European Organisation of Research and Treatment of Cancer Quality of Life Questionnaire Lung Cancer 13
FACT-L: Functional Assessment of Cancer Therapy-Lung
HRQOL: Health-related quality of life
MT: Maintenance therapy
NSCLC: Non-squamous non-small cell lung cancer
PROM: Patient-reported outcome measure
